# Importance of glycolysis and oxidative phosphorylation in advanced melanoma

**DOI:** 10.1186/1476-4598-11-76

**Published:** 2012-10-09

**Authors:** Jonhan Ho, Michelle Barbi de Moura, Yan Lin, Garret Vincent, Stephen Thorne, Lyn M Duncan, Lin Hui-Min, John M Kirkwood, Dorothea Becker, Bennett Van Houten, Stergios J Moschos

**Affiliations:** 1 Department of Dermatology, University of Pittsburgh, Pittsburgh, PA, 15213, USA; 2 Departments of Pharmacology and Chemical Biology, University of Pittsburgh, Pittsburgh, PA, 15213, USA; 3 Department of Biostatistics, University of Pittsburgh, Pittsburgh, PA, 15213, USA; 4 Department of Medicine, University of Pittsburgh, Pittsburgh, PA, 15213, USA; 5 Department of Surgery, University of Pittsburgh, Pittsburgh, PA, 15213, USA; 6 Department of Pathology, Massachusetts General Hospital, Boston, MA, 02114, USA; 7 Department of Pathology, University of Pittsburgh, Pittsburgh, PA, 15213, USA; 8 Present Address: Clinical Associate Professor, Department of Medicine, University of North Carolina at Chapel Hill Physicians Office Building, 3rd Floor, Suite 3116, CB #7305, 170 Manning Drive, Chapel Hill, NC, 27599, USA

**Keywords:** Melanoma, Lactate dehydrogenase, Glycolysis, Mitochondria, Oxidative phosphorylation, Monocarboxylate transporters

## Abstract

Serum lactate dehydrogenase (LDH) is a prognostic factor for patients with stage IV melanoma. To gain insights into the biology underlying this prognostic factor, we analyzed total serum LDH, serum LDH isoenzymes, and serum lactate in up to 49 patients with metastatic melanoma. Our data demonstrate that high serum LDH is associated with a significant increase in LDH isoenzymes 3 and 4, and a decrease in LDH isoenzymes 1 and 2. Since LDH isoenzymes play a role in both glycolysis and oxidative phosphorylation (OXPHOS), we subsequently determined using tissue microarray (TMA) analysis that the levels of proteins associated with mitochondrial function, lactate metabolism, and regulators of glycolysis were all elevated in advanced melanomas compared with nevic melanocytes. To investigate whether in advanced melanoma, the glycolysis and OXPHOS pathways might be linked, we determined expression of the monocarboxylate transporters (MCT) 1 and 4. Analysis of a nevus-to-melanoma progression TMA revealed that MCT4, and to a lesser extend MCT1, were elevated with progression to advanced melanoma. Further analysis of human melanoma specimens using the Seahorse XF24 extracellular flux analyzer indicated that metastatic melanoma tumors derived a large fraction of energy from OXPHOS. Taken together, these findings suggest that in stage IV melanomas with normal serum LDH, glycolysis and OXPHOS may provide metabolic symbiosis within the same tumor, whereas in stage IV melanomas with high serum LDH glycolysis is the principle source of energy.

## Introduction

It is now widely accepted that metabolic changes are one of the hallmarks of cancer [1]. The widespread use of Positron Emission Tomography (PET) imaging with 2-deoxyglucose (2-DG) uptake in several solid tumors supports the Warburg hypothesis, which posits that aerobic glycolysis is a major source of energy in malignant cells [2]. However, more recently, OXPHOS has been shown to also play a significant role in cancer metabolism [3-5]. Previous studies have documented that in melanoma, non-glycolytic pathways are important [6,7] and that oxygen consumption rate, a surrogate marker for mitochondrial respiratory chain activity, is one of the highest in human melanoma xenografts when compared with other tumor xenografts [8].

We recently showed by way of bioenergetics analysis that compared with melanocytes, metastatic melanoma cells propagated *in vitro* have elevated levels of OXPHOS, in addition to glycolysis [5]. Within the three-dimensional tumor where blood supply, and therefore oxygenation, can be variable, it has been proposed that its center, which is less oxygenated, is predominantly dependent on glycolysis, whereas the more vascularized tumor periphery is more dependent on OXPHOS. However these two spatially distinct populations can be metabolically linked such that lactate from the glycolytic portion of the tumor helps fuel ATP production in the vascularized region of the tumor through OXPHOS in a process termed metabolic symbiosis [9,10]. However, it is presently not known whether metastatic melanomas utilize these two crucial metabolic pathways in concert or sequentially.

Lactate dehydrogenase (LDH) has a central function in cellular metabolism and is comprised of five isoforms (LDH1-5). Each isoform is either a homotetramer (LDH1 and LDH5) or heterotetramer (LDH2, LDH3, and LDH4) of subunits encoded by the LDHA and LDHB gene (Additional file 1). Depending upon the LDH isoform and the concentration of pyruvate and lactate, the enzyme can interconvert these two compounds. More specifically, while LDH1 and LDH2 isoforms play a major role in the production of pyruvate from lactate, LDH4 and LDH5 are primarily involved in the production of lactate from pyruvate (17, 18). In the case of metastatic melanoma, it has been known for many years that approximately 30-40% of patients enrolled in randomized phase III clinical trials have high serum LDH, which correlates with poor prognosis [11]. Although to date, few randomized phase III melanoma trials have shown clinical benefit, post-hoc analysis of some trials, which overall were negative, did reveal statistically significant benefits in favor of the investigational arm for melanoma patients with normal versus high serum LDH [12-14]. The SYMMETRY study, a randomized phase III trial that determined efficacy of the small-molecule inhibitor Elesclomol, administered alone or in combination with paclitaxel, provided evidence that while the combination of Elesclomol with paclitaxel led to significant progression-free survival (PFS) in patients with normal serum LDH, there was a trend towards worse overall survival (OS) in patients with high serum LDH [15]. We [5] and others [16] have shown that Elesclomol suppresses OXPHOS in melanoma cells *in vitro*, and that melanoma cells without mitochondrial DNA (rho zero cells) are more resistant to low-to-moderate doses of Elesclomol. These findings, in addition to the critical role of LDH in the interconversion of lactate and pyruvate in the production of ATP through glycolysis or OXPHOS, prompted us to hypothesize that differences in serum LDH among patients with metastatic melanoma reflect differential dependence upon these bioenergetic pathways.

In view of these observations we sought to determine whether: 1) the LDH isoform expression profile and levels of lactate in serum obtained from up to 49 patients with metastatic melanoma would correlate with disease progression and OS; and 2) whether melanoma development and progression might be linked with key enzymes in glycolysis, OXPHOS, and lactate transport. Our data presented herein demonstrate that patients with advanced metastatic melanoma and high serum LDH have low levels of LDH1 and LDH2, but elevated levels of LDH3 and 4, suggesting that glycolysis is the primary metabolic pathway utilized by the tumor cells in these patients. In contrast, in patients with advanced melanoma and normal serum LDH, OXPHOS has an important role in addition to glycolysis for energy production.Furthermore, our data demonstrate that several key enzymes associated with high OXPHOS are substantially elevated in primary and metastatic melanomas compared with nevic melanocytes. Together these findings support a model that both glycolysis and OXPHOS play a significant role in developing metabolic symbiosis in metastatic melanoma progression.

## Materials and methods

### Patient sera, melanoma cell lines, and tumor cell suspensions

Sera from patients with stage IV metastatic melanoma were obtained in compliance with University of Pittsburgh Cancer Institute (UPCI) protocols 96–099 and 11–108. Overall survival (OS) was defined as the interval from collection of serum LDH, LDH isoenzyme, and serum lactate to death from any cause. Human epidermal melanocytes (HEMs) (Cell Applications, San Diego, CA) were propagated in melanocyte growth medium (Cell Applications). Human melanoma cell lines (WM983-A, WM983-B, WM1158, WM852, Lu1205, C32) were purchased from the Coriell Institute for Medical Research (Camden, NJ) or the American Type Culture Collection (Manassas, VA). The human melanoma cell line MV3 was obtained from Dr. S. Ferrone (University of Pittsburgh Medical Center), and the human melanoma cell line M233 was provided by Dr. A. Ribas (UCLA).The melanoma cell line TPF10-741, derived from a subcutaneous metastasis of a patient who developed secondary resistance to the BRAF inhibitor Vemurafenib, was established in compliance with UPCI protocol 96–099, as we have previously reported [5]. Single-cell suspensions from metastatic melanomas were also obtained in compliance with UPCI protocol 96–099.

### Antibodies

Antibodies used in the study were: LDHA (rabbit anti-human polyclonal, Abcam, Cambridge, MA); LDHB (mouse anti-human monoclonal, Sigma-Aldrich, St. Louis, MO); MCT1 and MCT4 (rabbit anti-human polyclonal, Chemicon, Temecula, CA); ATP synthase, H^+^ transporting, mitochondrial F1 complex, alpha subunit 1 (ATP5A1) (mouse anti-human monoclonal, Invitrogen, Carlsbad, CA); hypoxia-inducible factor 1 alpha (HIF-1α) (mouse anti-human monoclonal, BD Biosciences, San Diego, CA); and α-tubulin (rabbit anti-human monoclonal, Cell Signaling, Danvers MA).

### Immunoblot and TMA analysis

Melanoma cell lysates were analyzed by Western blots as previously described [17] to determine the performance of antibodies used as well as the relative expression levels of the corresponding proteins in melanocytes and various melanoma cell lines (Additional file 2: Figure S2). The previously described nevus>melanoma progression TMA [18] was probed with antibody and scored as previously described [19]. Briefly, using a 20X objective, the bright-field image of every antibody-probed tissue core was scored on a scale of 0 to 3+ (0 = no signal, 1+ = weak signal, 2+ = moderate signal, 3+ = strong signal). An H-score was calculated, which combined the intensity of the antibody staining-signal with the percentage of cells that exhibited an antibody signal at the different staining intensities [20]. H-scores were determined exclusively for melanoma cells.

### Measurement of total serum LDH, LDH isoenzymes, and lactate

All assays pertaining to total serum LDH, LDH isoenzymes, and lactate were performed by a clinical laboratory improvement and amendment (CLIA)-certified laboratory. This implies that, as per CLIA requirements, test results are provided with reference ranges of upper and lower limits of normal. More specifically, total serum LDH was measured as routine chemistry per manufacturer’s recommendations (Beckman Coulter, Inc., USA). Total serum LDH levels are presented as the ratio of each LDH measurement to the serum LDH value that is listed as the upper limit of normal (ULN). LDH isoenzymes were identified and quantitated by agarose gel electrophoresis on the SPIFE 2000/3000 Systems (Helena Laboratories, Beaumont, TX) [21]. Each of the five LDH isoenzymes is presented as percentage activity to total serum LDH activity. For lactate measurements, samples were maintained on ice at all times. Using lactate reagent (lactate oxidase, peroxidase, dichlorobenzenesulfonic acid, 4-aminoantipyrine), serum lactate was determined using the SYNCHRON Systems (Beckman Coulter, Inc., USA).

### Metabolic analysis

The metabolic profile of single-cell suspensions, prepared from ‘fresh’ metastatic melanoma tissue specimens, was determined using a Seahorse XF24 Extracellular Flux Analyzer (Seahorse Biosciences, Billerica, MA) and was performed on tissue specimens within six hours of surgery to remove metastatic melanoma tumors in compliance with UPCI protocol 96–099. The Seahorse Flux Analyzer provides real-time measurements of oxygen consumption rate (OCR), a measure of OXPHOS, and extracellular acidification rate (ECAR), a measure of glycolysis [5,22]. The single-cell suspensions, derived from four subcutaneous BRAF^V600E^-positive metastatic lesions from four different patients, were treated for 2 hr with collagenase IV (Worthington, Lakewood NJ). The melanoma cells were then attached for 30 minutes to tissue culture plates using Cell-Tak Cell and Tissue Adhesive (BD Biosciences). The cells were immediately analyzed in the Seahorse XF24 Extracellular Flux Analyzer under basal conditions (no treatment), and following injection of four pharmacologic inhibitors: Oligomycin (O) (1 μM), an inhibitor of ATP synthase, which allows a measurement of ATP-coupled oxygen consumption through OXPHOS; carbonyl cyanide 4-trifluoromethoxy-phenylhydrazone (FCCP) (300 nM), an uncoupling agent that allows maximum electron transport, and therefore a measure of maximum OXPHOS respiration capacity; 2-DG (100 mM), an inhibitor of glycolysis; and rotenone (R) (1 μM), an inhibitor of complex I of the mitochondrial respiratory chain that allows a precise measurement of mitochondrial uncoupling. All chemicals were obtained from Sigma-Aldrich.

### Statistical analysis

The R package for statistical computing software (version 2.11.1, http://www.R-project.org) was used for all statistical analyses. Pearson correlation coefficients (rho, ρ) were used to quantify the correlation between the percentage of each LDH isoenzymes/lactate levels and the *log*-transformed serum LDH levels. Fisher’s exact tests were used to determine possible correlations between LDH isoenzymes/lactate levels and serum LDH levels categorized as normal versus high. L*og*-rank tests were also performed to compare the OS between patients with high versus low total serum LDH, serum LDH isoenzymes, and serum lactate. Statistical analysis of the metabolism data was performed by one-way analysis of variance (ANOVA) followed by the Dunnett’s test.

Kruskal-Wallis (KW) tests were used to compare the level of expression of each molecule in the different stages of the nevus>melanoma progression TMA. Wilcoxon Rank-Sum tests were used to compare the expression level between two groups. Poisson regression models were fit to the expression level data of each molecule, and the likelihood-ratio test (LRT) was used to test the significance of the trend in the expression of each molecule across different stages of the nevus>melanoma progression TMA. Spearman’s correlation coefficients were calculated to quantify the association between the various molecules. The Bonferroni correction was used to account for multiple comparisons.

## Results

### Analysis of serum LDH isoenzymes in metastatic melanomas

**Figure 1:**
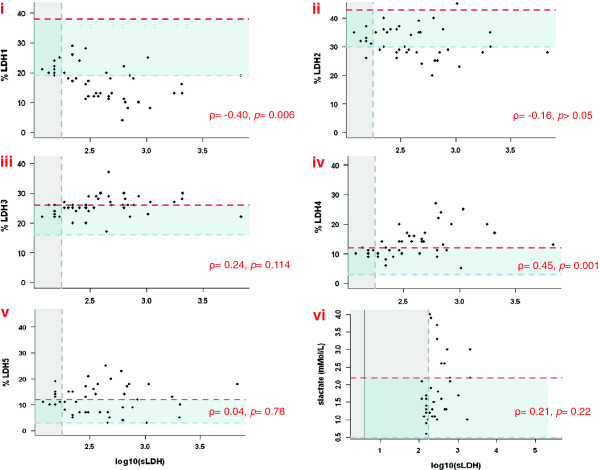
**Expression of serum LDH isoenzymes 1–5 and serum lactate in patients with metastatic melanoma.** Serum samples from melanoma patients analyzed for total serum LDH and LDH1-5 (panels i-v) and serum lactate (panel vi). Dots in each of the panels represent individual patient data showing the percentage of each LDH isoenzyme, presented as percentage activity to the total serum LDH activity, or absolute serum lactate levels versus *log*-transformed total serum LDH. Dashed blue and red lines in each panel depict the lower or upper limits of normal for each LDH isoenzyme percentage or serum lactate activity, respectively, whereas dashed grey lines depict normal and high total serum LDH levels. All lower and upper limits of normal are based on clinical cutoffs. Pearson’s correlation coefficients ρ, with corresponding *p*-values, determining the association between the percentage of each LDH isoenzyme and *log*-transformed total serum LDH are shown in red.

Of the five tetrameric LDH isoenzymes, LDH1 and LDH2 are preferentially associated with conversion of pyruvate from lactate for use in OXPHOS, while LDH4 and LDH5 are involved in the production of lactate in glycolysis (Additional file 1) [23]. The levels of serum LDH1-5 isoenzymes and serum lactate in up to 49 patients with metastatic melanoma were determined. As shown in Figure 1, panel i-v, for isoenzymes 1 thru 5, the percentage of LDH1 was negatively associated with the *log*-transformed total serum LDH level. However, a positive association was observed between the percentage of LDH4 and *log*-transformed total serum LDH level. In contrast, the percentage of LDH5, the most efficient isoform in catalyzing conversion of pyruvate to lactate, did not correlate with log-transformed serum LDH levels. To further determine a possible correlation between high serum LDH and high serum lactate, we subsequently measured lactate levels in the patients’ sera. As shown in Figure 1, panel vi, serum lactate levels show a weak insignificant association with the *log*-transformed serum LDH levels. We further categorized each of the LDH isoenzymes, total LDH level, and serum lactate level into low, normal, and high levels using the clinical cutoffs. Abnormally low levels of LDH 1 and 2 are associated with abnormally high serum LDH level (Fisher’s exact test *p* = 0.0001 and *p* = 0.047) and abnormally high levels of LDH 3 and 4 are associated with abnormally high serum LDH level (*p* = 0.011 and *p* = 0.0005). Again, no association was detected between the high LDH5 and high serum LDH levels. To assess whether any of the serum LDH isoenzymes or serum lactate correlated with OS we performed a survival analysis. At a median follow-up of 5.8 months (range 0.07-17.8 months) 31 out of 49 patients had died. As previously shown [11], patients with a more than 1.2-fold increase in total serum LDH levels had shortened OS compared with patients with an equal or less than 1.2-fold increase in total serum LDH (log-rank *p* = 0.015). Furthermore, patients with low serum LDH1 or high serum LDH4 had a worse OS compared with patients with normal serum LDH1 and 4 (*p* = 0.057 and *p* = 0.019, respectively).

### Expression of LDHA and HIF-1Î± in the nevus>melanoma progression pathway

**Figure 2:**
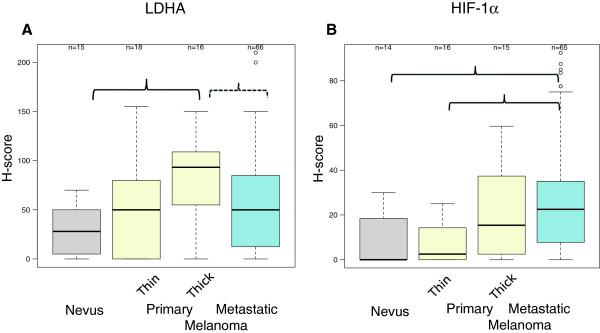
**LDHA and HIF-1α expression in nevi and melanomas.** Boxplots (median with the 25^th^ and 75^th^ percentiles) of LDHA (panel **A**) and, likewise, HIF-1α (panel **B**) H-scores of the TMA cores representing nevi, thin primary melanoma, primary thick melanoma, metastatic melanoma (visceral and lymph node metastases). Depicted in each case is the mean of the average of replicate spots with the same histologic diagnosis. Solid bracket lines depict significant (KW test p≤0.05), and the dashed bracket line (panel **A**) indicates trend for significant (KW test 0.05<p<0.1) pairwise comparisons corrected for multiple comparison testing. Outliers in the analysis are indicated by open circles.

The detected changes in the LDH isoenzymes, and, in particular, the increase of the more glycolytic LDH isoenzymes (LDH3 and LDH4) along with the corresponding decrease of the non-glycolytic LDH isoenzymes (LDH1 and LDH2), may be attributable to changes in expression of the LDHA and LDHB subunits in melanoma cells. To investigate whether melanoma cells express LDHA that mediates conversion of pyruvate to lactate, we performed immunohistochemistry studies of a nevus>melanoma progression TMA. As shown in Additional file 3 LDHA is expressed in nevic melanocytes, and in primary and metastatic melanomas, but its levels are higher in primary thick melanomas. Depicted in Figure 2, panel a, are boxplots of the TMA LDHA data, which document increased expression of LDHA with progression from nevi to advanced melanoma (Poisson model LRT *p*<0.001). Pairwise comparison between different stages of the nevus>melanoma progression pathway showed that the most significant increase was between primary thick primary melanomas and nevi, with the mean LDHA expression increased approximately three-fold (Wilcoxon adjusted *p*=0.003). In addition, the data also indicate trend towards reduction between thick primary melanomas and metastatic melanomas.

Since it has been reported that HIF-1α increases LDHA expression [24], we also determined HIF-1α expression in the nevus>melanoma TMA. In agreement with the data of a previous report [25], HIF-1α was expressed in primary melanoma tissues (Additional file 3). Figure 2, panel b, demonstrates that HIF-1α expression increased with progression from nevi to advanced melanoma —approximately 4-fold increase in mean HIF-1α expression between thin primary and metastatic melanomas, and especially lymph node metastases. However, overall expression was low and somewhat heterogeneous, especially in metastatic melanoma tissue cores (H-score < 100; Poisson model LRT *p* < 0.001). The significant differences in HIF-1α expression between nevi and melanomas were consistent with the data of an immunoblot analysis of cell lysates obtained from cultured HEMs, the radial growth phase melanoma cell line WM983-A, and various metastatic melanoma cell lines. In particular, HIF-1α was nearly absent in HEMs, present but at very low levels in the vertical growth phase WM983-A melanoma cell line, and various metastatic melanoma cell lines including WM983-B, which along with the WM-983-A were obtained from the same patient (Additional file 2).

### Involvement of OXPHOS in melanoma progression

**Figure 3:**
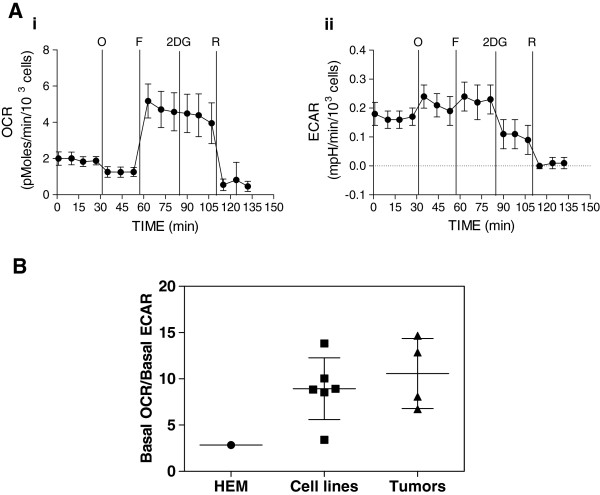
**Bioenergetics analysis in single-cell melanoma suspensions, melanoma cell lines, and HEMs.A**. Single-cell suspensions from a ‘fresh’ tumor respond to pharmacologic inhibitors of metabolism, as determined by a Seahorse XF24 Extracellular Flux Analyzer. Depicted are OCR (panel i, left) and ECAR (panel ii, right) rates of single-cell suspensions, prepared from one of four metastatic melanomas. Similar results were obtained with the other 3 tumors. After baseline OCR and ECAR determination, the melanoma cells were treated with oligomycin (O), FCCP (F), rotenone (R), or 2-DG. **B**. Ratio of basal OCR over basal ECAR in HEMs, melanoma cell lines, and single-cell melanoma suspensions. Data are depicted as the ratio of basal OCR over basal ECAR ratio of short-term cultures of HEMs, six different metastatic human melanoma cell lines (WM983-A, WM983-B, WM1158, WM852, Lu1205, and C32), and single-cell suspensions obtained from four subcutaneous metastatic melanomas.

We previously reported that compared with HEMs, melanoma cells are more metabolically active, and in addition to glycolysis, derive a significant proportion of energy from OXPHOS [5]. Using a Seahorse XF24 extracellular flux analyzer, which provides real-time measurements of oxygen consumption rate (OCR), a marker of OXPHOS, and extracellular acidification rate (ECAR), a surrogate of glycolysis, we determined both the basal rates and reserve capacity of OXPHOS in tumor cells freshly isolated (within six hrs) from tumor biopsies derived from four patients with metastatic melanoma. Representative data for one of these patient samples are shown in Figure 3A. After measuring the basal rates of OCR and ECAR, pharmacologic inhibitors of the mitochondrial respiratory chain (oligomycin, FCCP, rotenone) and of glycolysis (2-DG) were injected to determine both the amount of ATP being derived from OXPHOS (oligomycin-inhibitable) and the reserve capacity of OXPHOS (FCCP-induced) in the tumor cells. Of note, melanoma cells derived from this particular patient exhibited a high reserve capacity for OXPHOS, as indicated by the large increase in OCR after FCCP injection (Figure 3A, panel i). To directly compare the data from these four patient samples to the data derived from the melanoma cell lines propagated *in vitro*, we measured the ratio of the basal OCR (Figure 3A, panel i) to basal ECAR (Figure 3A, panel ii), using the equation:

(1) $$\frac{pMoles}{\frac{\min}{1,000}cells}/\frac{mpH}{\min/1,000cells}=\frac{pMoles}{mpH}$$

Figure 3B documents that the mean OCR/ECAR ratio of melanoma cells derived from the four tumor biopsies was not significantly different from the OCR/ECAR ratios of six melanoma cell lines propagated in culture. However, the OCR/ECAR ratio of both freshly isolated melanoma tumor cells and melanoma cell lines was higher than the mean OCR/ECAR ratio of HEMs. Together these data suggest that in tumors representing metastatic melanomas a substantial proportion of energy is derived from OXPHOS while HEMs propagated in vitro have lower OXPHOS and higher glycolysis.

**Figure 4:**
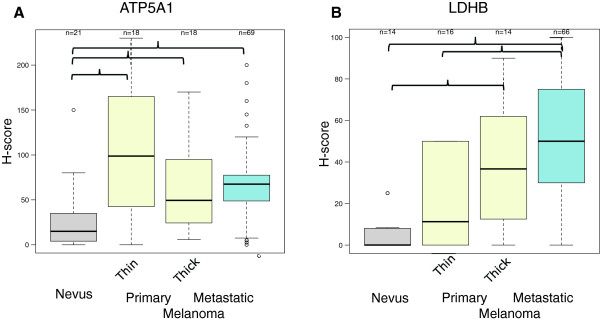
**ATP5A1 and LDHB expression in nevi and melanomas.** Boxplots of ATP5A1 or LDHB expression in the different histologic stages of nevus>melanoma progression. Depicted is the mean of replicate spots with the same histologic diagnosis. Solid brackets illustrate significant (KW test p≤0.05) pairwise comparisons corrected for multiple comparison testing. Outliers in the analysis are indicated by open circles.

To obtain additional experimental evidence that OXPHOS is elevated in melanoma cells, we used an antibody to ATP5A1, a mitochondria-encoded subunit of ATP synthase [26], as a ‘marker’ for OXPHOS to probe the nevus>melanoma progression TMA. In addition, we used an antibody to LDHB, which encodes all four subunits of LDH1 that converts lactate to pyruvate. ATP5A1 expression was detected prevalently in the cytoplasm of melanoma cells, but some expression was also seen in tumor-interspersing macrophages and, in some cases, in the stratum spinosum and stratum basale layer of the epidermis (Additional file 4: Figure S4A). Like in the case of ATP5A1, LDHB expression was detected predominantly in the cytoplasm of melanoma cells (Additional file 4). LDHB, but not ATP5A1, expression was significantly increased in advanced melanomas compared with nevi (Poisson model LRT *p* < 0.001 and *p* = 0.098, respectively). However, the mean ATP5A1 expression was increased by at least 2.5-fold in metastatic melanomas compared with nevi. The mean increase in LDHB expression was even higher, and, in particular between nevi and metastatic melanomas (approximately 10-fold; Figure 4). Given the nature of immunohistochemical analysis of TMA, we would like to point out that we could not address whether the observed increase in ATP5A1 expression is the result of increased numbers of mitochondrial and/or increased numbers of components in the mitochondrial respiratory chain of the individual mitochondria. However, these findings and the TMA data described below are in agreement with the findings of our bioenergetics studies presented herein (Figure 3) as well as those we previously reported [5], which have shown that mitochondrial respiration is an important biologic feature of advanced melanomas.

### Role of MCTs, indirect regulators of metabolism, in melanoma

**Figure 5:**
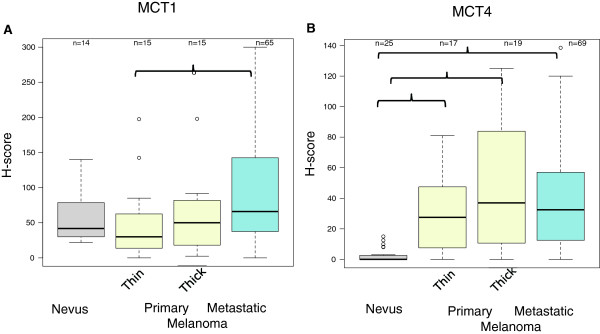
**MCT1 and MCT4 expression in the nevus>melanoma TMA.** The TMA study was performed and data were analyzed as described in the legends to Figures 2 and 4. Solid bracket lines show significant (KW test p ≤ 0.05) pairwise comparisons corrected for multiple comparison testing.

The data presented suggest that in addition to glycolysis, OXPHOS is important for melanoma progression. Mouse xenograft models have shown that cancer cells that are located in different areas of the tumor preferentially utilize one metabolic source over the other depending on environmental cues [10]. Given that lactate produced by glycolytic cells can be taken up by OXPHOS-dependent cancer cells via MCTs under an ATP-independent passive diffusion that follows substrate gradients and H^+^ transport [10] it is a possibility that disrupting MCT function might be catastrophic for cells that are dependent on glycolysis, for the reason that the cells may not be able to release lactic acid, resulting in lowering the pH and death [27]. Conversely, MCT inhibition may limit carbon source supply to OXPHOS-utilizing cancer cells that are in part dependent on lactate released by glycolytic cells for their metabolism. In fact, a previous study suggested that targeting MCT1 and 4 might be a particularly effective approach against melanoma *in vivo*[28]. Given the lack of information regarding expression of MCT1 and MCT4 in melanoma tumor tissues and the availability of highly specific small molecule MCT inhibitors, several of which are in early clinical development (e.g. CRUKD/12/004; *a phase I study of AZD3965, an MCT1 inhibitor, in advanced MCT1-expressing cancers*), we became interested in studying the expression of MCT1 and MCT4 in the nevus>melanoma TMA. Our TMA analysis revealed that MCT1 expression was primarily membranous while MCT4 expression was mostly cytoplasmic (Additional file 5). Figure 5 demonstrates that expression of both MCT1 and MCT4 increased with progression from nevi to advanced melanoma (Poisson model LRT *p*<0.001 for both MCT1 and MCT4). Furthermore, pairwise analysis showed that the mean increase in MCT4 expression was significant in both primary melanomas (thin and thick) and metastatic melanomas compared with nevi (at least 12-fold), but not between primary and metastatic melanomas. On the other hand, MCT1 expression was elevated significantly only in metastatic melanomas when compared with thin primary melanomas (KW *p*=0.036). The immunohistochemical data are in agreement with the data from the immunoblot analysis of MCT1 and MCT4 expression in cultured HEMs and melanoma cell lines (Additional file 2), which revealed lack of expression of both MCT1 and MCT4 in HEMs, frequent expression of MCT1 versus MCT4 in melanoma cell lines, and a positive correlation between HIF-1α and MCT4 expression in different melanoma cell lines (MV3, WM852, WM983-A, WM983-B, ATCC72, C32).

To explore a possible link between proteins involved in glycolysis (LDHA, HIF-1α), OXPHOS and mitochondrial function (ATP5A1, LDHB), and lactate metabolism and transport (LDHA, LDHB, MCT1, MCT4), we used Pearson correlation analysis between each permutation pair of the datasets of the respective proteins. The results of this analysis (Additional file 6) revealed a moderate (0.3<ρ≤0.42), albeit significant link (*p*<0.001) between HIF-1α and LDHA, and between HIF-1α and MCT4, but not between ATP5A1 and HIF-1α, or MCT1 and MCT4. The analysis also revealed significant associations between OXPHOS (ATP5A1 and LDHB, MCT1 and LDHB), glycolysis (MCT4 and LDHB), and OXPHOS and glycolysis (LDHA and LDHB).

## Discussion

For more than two decades, ^18^F-2-DG has been used for diagnostic purposes as a contrast agent for melanoma, and high serum LDH as a prognostic factor in metastatic melanoma [29]. However, not much is known regarding the importance of crucial metabolic pathways in melanoma development and progression. With this present study we have obtained the following novel insights. First, we demonstrate that patients who have high serum LDH levels have elevated levels of LDH isoenzymes, which drive pyruvate conversion to lactate. Second, enzymes associated with glycolysis, as well as OXPHOS, are expressed at higher levels in primary and metastatic melanomas than in nevi, which suggests a correlate between progression to advanced melanoma and increased metabolic flexibility. Third, this is the first study to directly measure the relative contribution of OXPHOS and glycolysis in melanoma cells directly from patients, which showed that OXPHOS plays an important role in the generation of ATP in melanoma cells. Finally, the data presented herein document that key regulators of lactate transport and pH are differentially expressed in melanomas compared with nevi.

Our finding that patients with advanced melanoma have elevated levels of serum LDH3 and LDH4, but reduced levels of serum LDH1 and LDH2, has also been reported in the case of breast cancer and other solid malignancies [30,31]. However, it is not yet completely understood what accounts for this LDH isoenzyme pattern. We postulated that changes in the expression levels of individual LDHA and B isoenzymes account for changes in the LDH isoenzyme pattern. Data from our nevus>melanoma progression TMA analysis, suggest that metabolic changes in melanoma, but not in adjacent stromal, cells might induce this differential serum LDH isoenzyme profile. This conclusion is based on the finding that LDHA was elevated in primary melanomas compared with nevi, and was even more elevated in thick (Breslow thickness of invasion > 2 mm) primary melanomas. In addition, the significant correlation between LDHA and HIF-1α expression we detected in our TMA analysis is in agreement with previous studies, which showed that HIF-1α increases glycolytic metabolism, including the upregulation of LDHA [24]. The absence of increased LDH5 in patients with high serum LDH can be explained by the significant positive correlation between LDHA and LDHB expression in melanomas (Additional file 6) and that more LDHB than LDHA proteins are upregulated in advanced melanomas (Figures 2 and 4). The lower expression of LDH5 in metastatic compared with thick primary melanoma can be explained by intra-tumor heterogeneity in advanced melanoma cases in terms of reliance of melanoma cells to glycolysis versus OXPHOS. In other words, several tumor cores that comprised this TMA may have been obtained from specific regions of the tumor that were metabolically dissimilar. In fact, one third of the tumor cores were derived from lymph node metastatic melanoma. Unfortunately, the lack of clinicopathologic annotation makes it impossible to address the significant variability in LDHA expression seen in metastatic melanoma.

We [5] and others [32] have previously found that in the case of melanoma, OXPHOS plays an important role in the cells’ metabolism in addition to glycolysis *in vitro*. Data from our study including: 1) the bioenergetics analysis in melanoma cells derived directly from patients; 2) the immunohistochemical analysis of ATP5A1, a key regulatory component of the mitochondrial respiratory chain; and 3) high expression of LDHB, a key enzyme that converts lactate to pyruvate, all support the importance of OXPHOS in melanoma. These data, which are in agreement with two previous reports in melanoma [8,33] do not necessarily refute the Warburg hypothesis that cancer cells rely heavily upon glycolysis for energy production [2]. Instead, our data strongly suggest that the metabolically flexibility of melanoma cells can provide selective advantage for tumor cell survival in diverse environments with low oxygen tension, scarce carbon source availability, and low pH [9].

In melanoma, monocarboxylate transporters should be added to the increasing complexity of cancer metabolism resources/pathways, which include glycolysis, reductive carboxylation of glutamine [6,34], and OXPHOS [5]. MCT1 and MCT4 play an important role in the transport of carbon sources among cancer cells in relation to environmental cues, such as pH and oxygen tension. The observed significant upregulation of MCT4, the principal transporter for lactate efflux [35] in melanomas compared with nevi may reflect conditioning of melanoma cells to high rates of lactate production that must be exported out of cells for survival. Our data point to a positive correlation between the immunohistochemical expression of MCT4 and HIF-1α and MCT4 and LDHA, which is in agreement with previous reports that MCT4 is a downstream effector of HIF-1α [36]. Together these data suggest that MCT4 may be expressed by the fraction of melanoma cells in the tumor that predominantly utilizes glycolysis for energy production and may be a critical therapeutic target.

**Figure 6:**
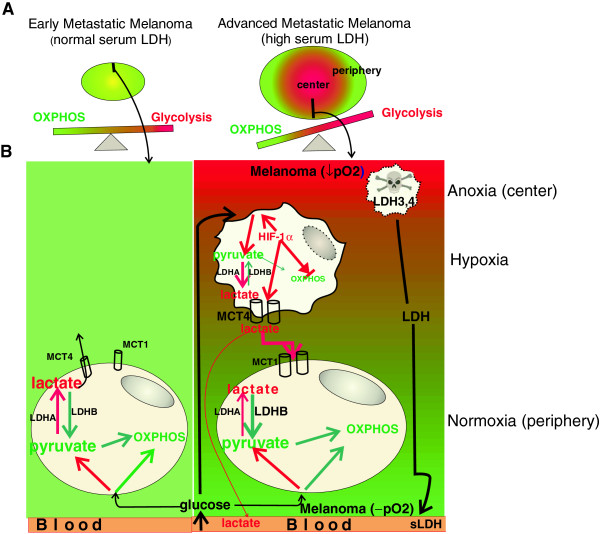
**Glycolysis and OXPHOS balance in metastatic melanoma.** (**A**) The cartoon serves to illustrate our hypothesis that in melanoma patients with normal serum LDH, equilibrium exists between glycolysis and OXPHOS, whereas in melanoma patients with high serum LDH, this balance shifts more towards glycolysis. In the center (indicated by the color red) of a melanoma that is more distant from the blood supply (indicated by the color green), the resulting hypoxia and thus, upregulation of a HIF-1α-dependent glycolytic program is taking up a larger portion of the tumor. (**B**) In patients with metastatic melanoma and normal serum LDH, glycolysis (indicated by red arrows) and OXPHOS (indicated by green arrows) are both utilized by melanoma cells. Excess H^+^ due to lactate production from glycolysis is extruded predominantly by MCT4. In larger size tumors from patients with metastatic melanoma and high serum LDH, two additional populations are emerging due to increasing distance of the tumor’s center from blood supply.

The lack of substantial upregulation of serum lactate levels in patients with high serum LDH raises the question whether the lactate that is produced by glycolytic melanoma cells is taken up by metabolically symbiotic tumor cells deriving their energy from OXPHOS before reaching the systemic circulation, which is in support of this metabolic symbiosis model. These data are also in agreement with a previous report, which showed that both glycolysis- and OXPHOS-dependent tumor cells regulate their access to energy metabolites [10]. Our study also suggests that advanced melanomas become glycolytic due to their hypoxic state, which might be linked to hypoxia-induced stabilization of HIF-1α activity. Although HIF-1α regulates the glycolytic program, it also suppresses OXPHOS [37]. Based upon our data and those of a previously published study [10], we depict a cartoon (Figure 6) that serves to illustrate a working model for metabolic requirements in melanoma. To meet the high metabolic ‘demands’ associated with melanoma development, proliferation, migration, and "invasion, both OXPHOS and glycolysis are increased in advanced melanoma compared with nevi, which are likely to be metabolically less active tissues. In addition to the balanced upregulation of these two metabolic pathways, advanced melanoma cells acquire the ability to utilize non-glucose sources, such as lactate (LDHA and MCT1 upregulation), as well as the ability to prevent buildup of acid that may prevent tumor growth (MCT4 upregulation). In metastatic melanomas with normal serum LDH, balanced reliance on both OXPHOS and glycolysis is in place until the tumors grow beyond a certain size and thereupon, hypoxic regions develop (metastatic melanomas with high serum LDH). At this point in tumor progression, three distinct melanoma cell populations evolve: one that resides in extremely hypoxic areas, is necrotic, and releases glycolytic LDH isoenzymes (LDH3, 4), which is the primary cause for increased serum LDH. The second melanoma cell population resides in hypoxic areas, upregulates the HIF-1α glycolytic program, and releases lactate and hydrogen ions to maintain normal pH. The third population is in well-oxygenated areas and dependent upon OXPHOS by metabolizing glucose as well as lactate, which is released from the nearby glycolytic melanoma cell population. The mechanism(s) that tip(s) the balance towards more glycolysis and less OXPHOS in advanced melanoma are unclear, but may involve, as Otto Warburg described, mitochondrial dysfunction. This loss in mitochondrial function may be “functionally reversible” (i.e. hypoxia) or “permanently irreversible” (i.e. mutations in the mitochondrial DNA that encode for components of the mitochondrial respiratory chain) [38,39].

Finally, we believe that the findings of our study may be of relevance with respect to the design of future melanoma trials. First, the association of low LDH1 isoenzyme levels, in addition to high LDH4 levels with OS in patients with high serum LDH implies that changes in the reliance of both OXPHOS and glycolysis, but not only glycolysis alone, are clinically important. Therefore, agents that attempt to revert the reliance of cancer cells more on OXPHOS and less on glycolysis may be clinically effective [40]. Second, since serum LDH levels define two different metastatic melanoma subgroups, one with normal serum LDH levels that is primarily dependent upon OXPHOS and the other with high serum LDH that is predominantly comprised of cells that are dependent upon OXPHOS, therapies that only disrupt OXPHOS may only have an effect in patients with normal serum LDH [5]. One such therapy is Elesclomol that we [5] and others [16] have shown to suppress OXPHOS by disrupting components of the mitochondrial respiratory chain that was previously shown to have an opposite clinical effect in patients with metastatic melanoma and normal versus high serum LDH [15]. On the other hand, patients with high serum LDH bear melanomas with hypoxic areas that produce high HIF-1α levels which upregulate components of the glycolytic pathway in addition to components of the angiogenesis pathway, such as the vascular endothelial growth factor. These tumors are expected to be more prone to glycolysis inhibitors, yet to be identified, as well as to angiogenesis inhibitors, such as bevacizumab, as previously described [14]. The findings of our study also suggest that MCT4, which transports lactate from glycolytic cells, has an important, and most likely, equally important role alongside MCT1. However, targeting MCT4 may clinically be more effective when melanomas become MCT1 independent, or when targeted in combination with MCT1 to prevent resistance to treatment [41].

## Competing interests

The authors declare that they have no competing interests.

## Authors’ contributions

JH, SJM, and LMD performed immunohistochemical analysis of the nevus-melanoma TMAs. MBM and GV performed Seahorse analysis of melanoma cell lines and tumor tissues. GV established melanocytes and melanoma cell cultures and immunoblot analysis for each of the antibodies used for immunohistochemical analysis. ST, GV, MBM performed *in vivo* imaging of glycolysis of human melanoma xenografts that supported the notion of metabolic symbiosis (data not shown). YL and LHM performed analysis of serum LDH and immunohistochemical data. SJM and JMK provided patient samples (sera and tumor tissues). ST, JMK, DB, BVH and SJM analyzed all experiments and wrote the manuscript. All authors read and approved the final manuscript.

## Supplementary Material

Additional file 1 ** Figure S1.** Schematic presentation of LDH1-5 and their involvement in OXPHOS and glycolysis. Red circles indicate LDHA subunits and blue circles indicate LDHB subunits. Click here for file

Additional file 2 ** Figure S2.** Validation of antibodies used in the nevus-melanoma TMA analyses. Immunoblot analysis of whole cell lysates, prepared from HEMs and different melanoma cell lines were probed with antibody specific for MCT4, MCT1, HIF-1α, LDHB, LDHA. α-tubulin served as loading control. Click here for file

Additional file 3 ** Figure S3.** LDHA and HIF-1α expression in nevi and melanomas. (A-B, panels a) TMA cores comprised of nevi, and primary and metastatic melanoma tissue core, probed with antibody to LDHA or HIF-1α, and counterstained with hematoxylin. (A-B, panels b) 10X magnification of select TMA cores. Click here for file

Additional file 4 ** Figure S4.** ATP5A1 and LDHB expression in nevi and melanomas. (A-B, panels a) TMA cores comprised of nevi, primary melanoma, and metastatic melanoma, probed with antibody to ATP5A1 or LDHB, and counterstained with hematoxylin. (A-B, panels b) 10X magnification of select TMA cores. Click here for file

Additional file 5 ** Figure S5.** MCT1 and MCT4 expression in the nevus>melanoma TMA. The TMA study was performed as described in the legends to Additional files 3 and 4: Figures S3 and S4. Click here for file

Additional file 6 ** Figure S6.** Pairwise correlation matrix analysis of expression of the various molecules in the nevus>melanoma TMA. Depicted in the lower left corner are dot plots of the H-scores between each permutation pair of the dataset of the six proteins whose expression was determined in the TMA. Shown in the upper right corner are Spearman rank correlation coefficients with corresponding *p*-values for the respective permutation pair of the six proteins. Rho and p values showing significant association are highlighted, and/or presented in a larger font size. Green-colored boxes in the matrix depict expected and known significant associations, blue-colored boxes show known insignificant associations, and red-colored boxes denote novel and significant associations. Click here for file
