# Supplementary figures and images for: Importance of glycolysis and oxidative phosphorylation in advanced melanoma
Source: Mol Cancer. 2012 Oct 9;11:76. doi: 10.1186/1476-4598-11-76 (PMC3537610; doi:10.1186/1476-4598-11-76)

## Slide 1
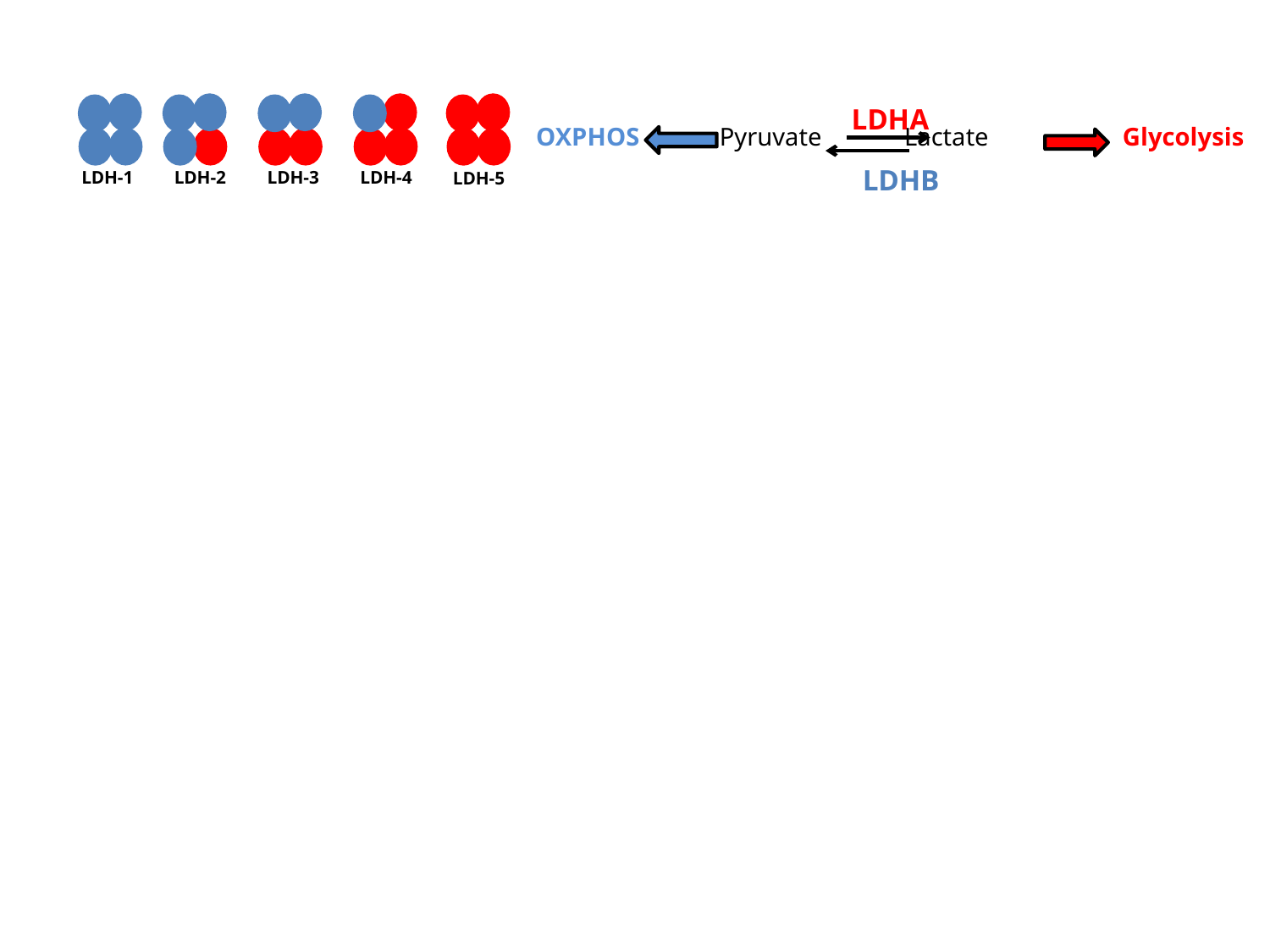

LDH-1
LDH-2
LDH-3
LDH-4
LDH-5
LDHA
OXPHOS
Pyruvate Lactate
Glycolysis
LDHB

Supplement: Additional file 1 — Figure S1. Schematic presentation of LDH1-5 and their involvement in OXPHOS and glycolysis. Red circles indicate LDHA subunits and blue circles indicate LDHB subunits. [file 1476-4598-11-76-S1.pptx]

## Slide 1
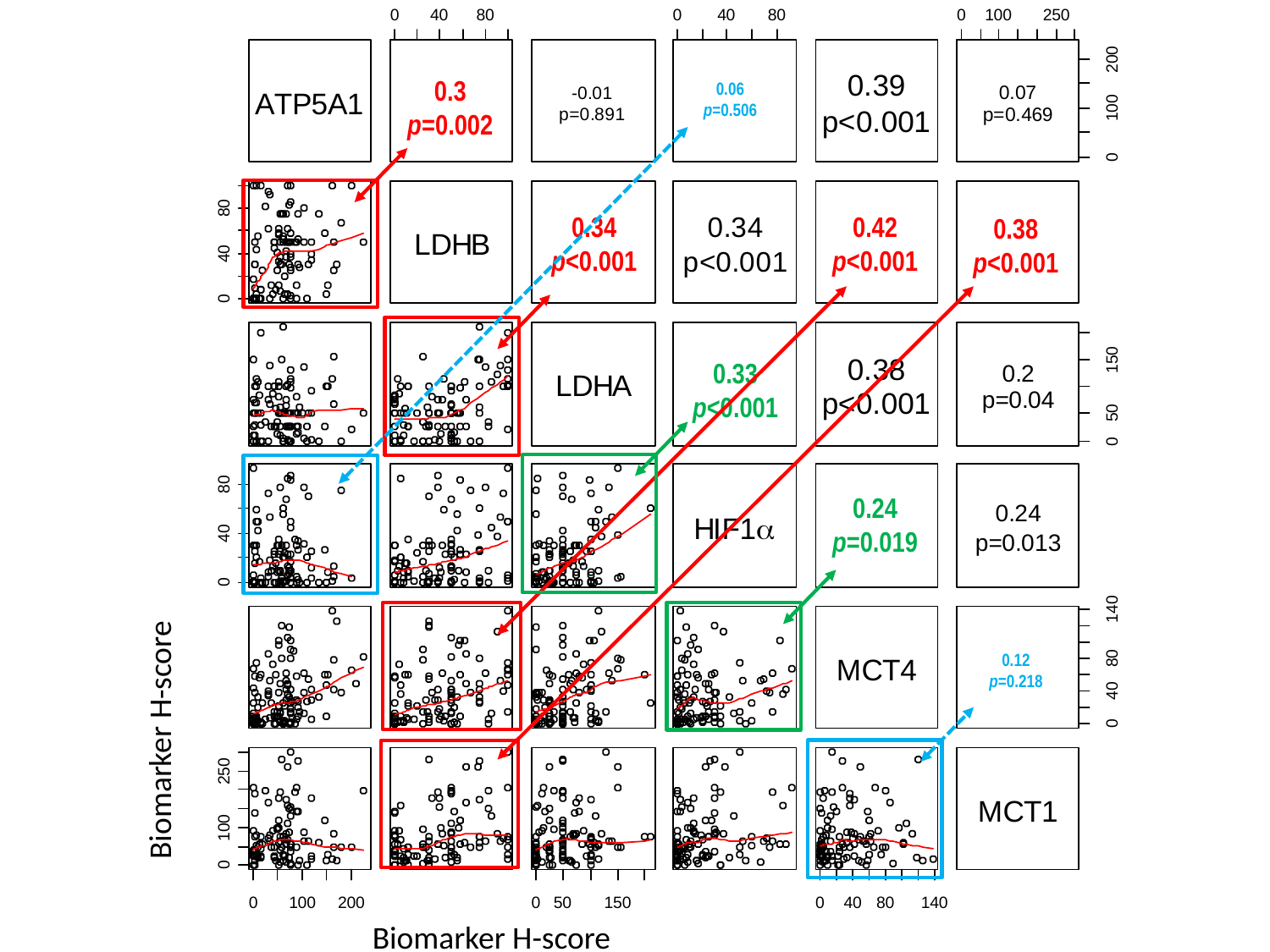

0.3
p=0.002
0.06
p=0.506
0.34
p<0.001
0.42
p<0.001
0.38
p<0.001
0.33
p<0.001
0.24
p=0.019
0.12
p=0.218
Biomarker H-score
Biomarker H-score

Supplement: Additional file 6 — Figure S6. Pairwise correlation matrix analysis of expression of the various molecules in the nevus>melanoma TMA. Depicted in the lower left corner are dot plots of the H-scores between each permutation pair of the dataset of the six proteins whose expression was determined in the TMA. Shown in the upper right corner are Spearman rank correlation coefficients with corresponding p-values for the respective permutation pair of the six proteins. Rho and p values showing significant association are highlighted, and/or presented in a larger font size. Green-colored boxes in the matrix depict expected and known significant associations, blue-colored boxes show known insignificant associations, and red-colored boxes denote novel and significant associations. [file 1476-4598-11-76-S6.pptx]
