# Supplementary material for: Importance of glycolysis and oxidative phosphorylation in advanced melanoma
Source: Mol Cancer. 2012 Oct 9;11:76. doi: 10.1186/1476-4598-11-76 (PMC3537610; doi:10.1186/1476-4598-11-76)
Supplement: Additional file 2 — Figure S2. Validation of antibodies used in the nevus-melanoma TMA analyses. Immunoblot analysis of whole cell lysates, prepared from HEMs and different melanoma cell lines were probed with antibody specific for MCT4, MCT1, HIF-1α, LDHB, LDHA. α-tubulin served as loading control. [file 1476-4598-11-76-S2.pptx]

## Slide 1
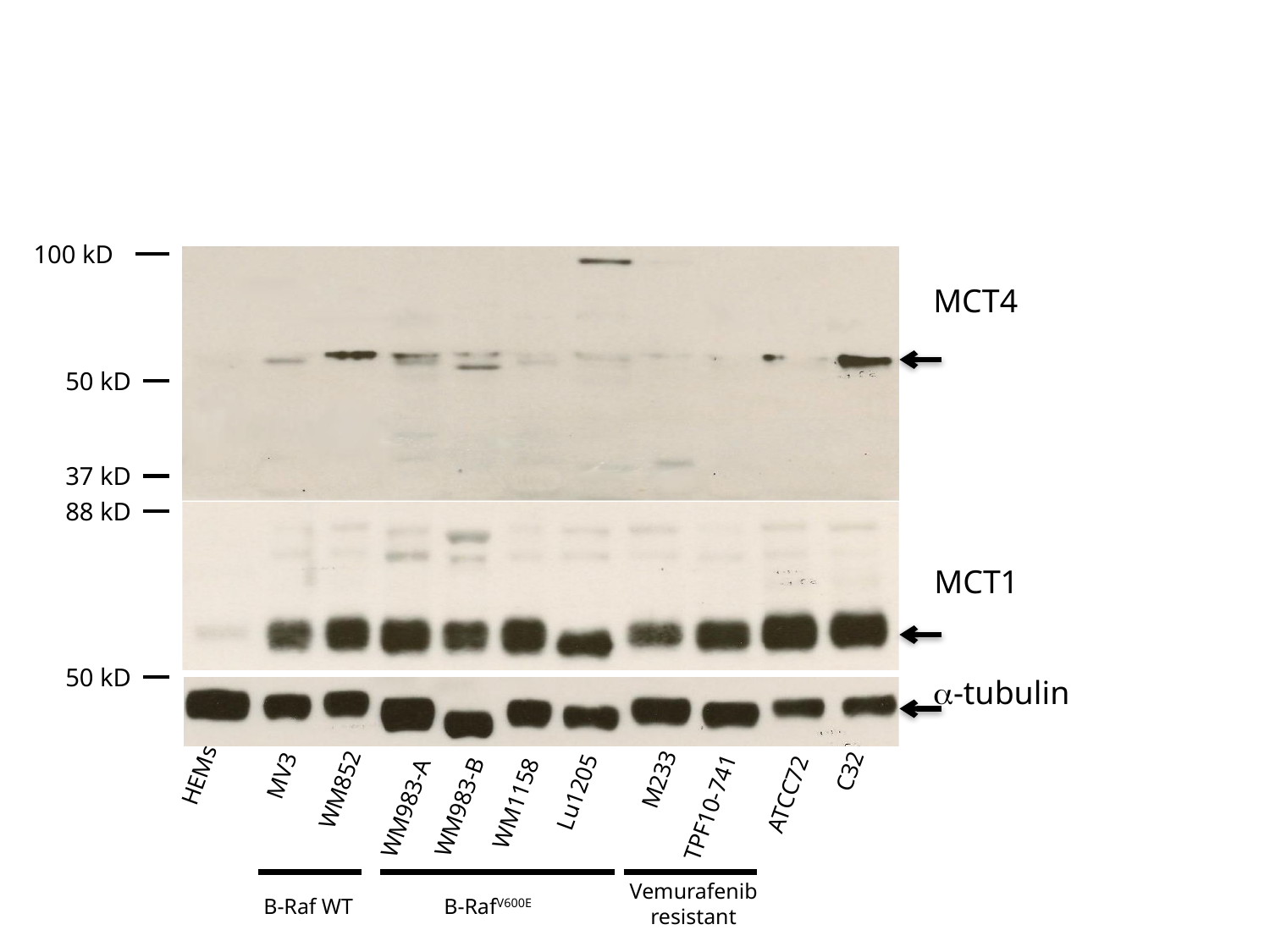

100 kD
MCT4
MCT1
α-tubulin
50 kD
37 kD
88 kD
50 kD
MV3
M233
HEMs
WM852
ATCC72
Lu1205
WM1158
TPF10-741
WM983-A
WM983-B
C32
Vemurafenib resistant
B-Raf WT
B-RafV600E

## Slide 2
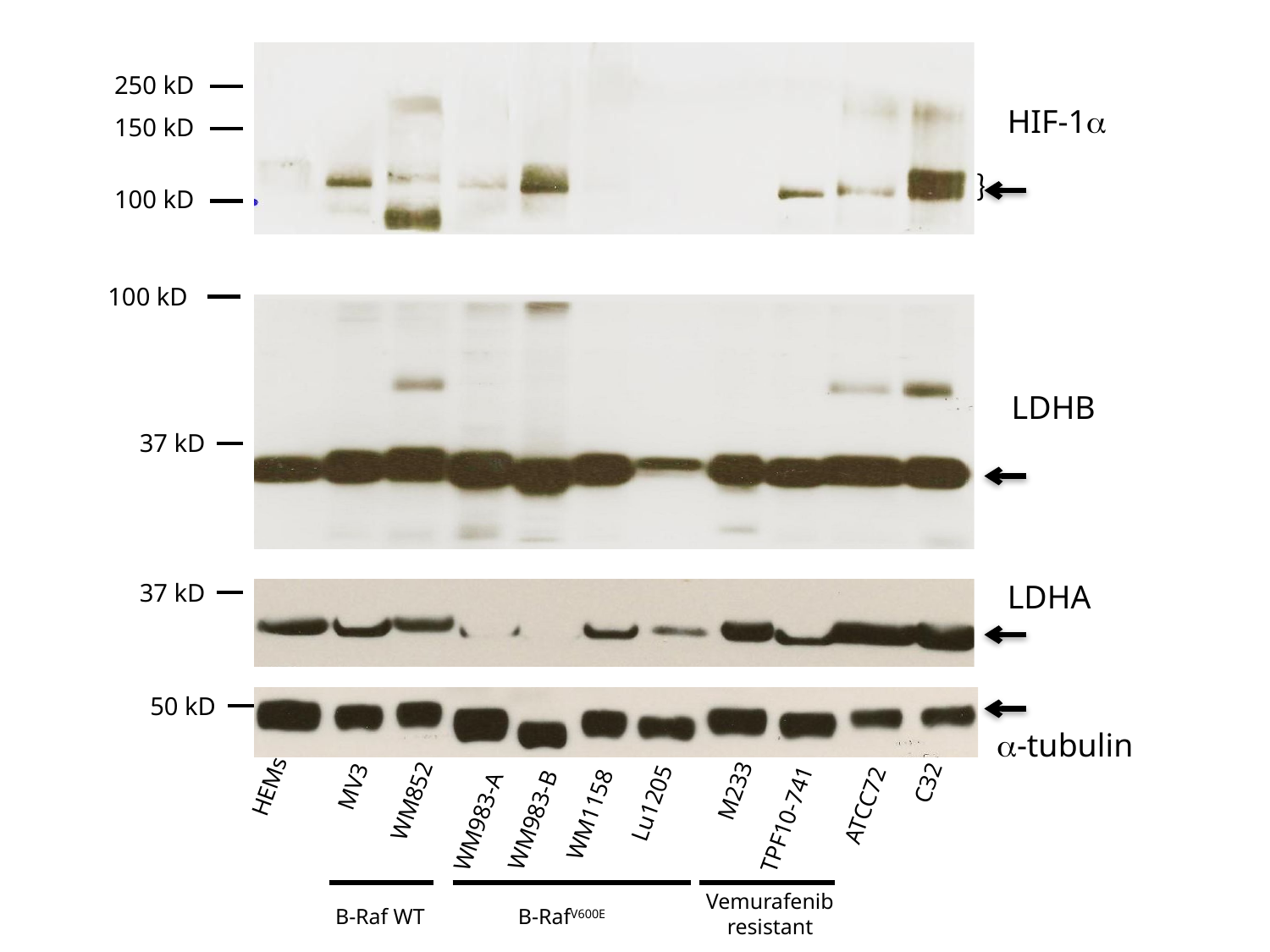

HIF-1α
250 kD
150 kD
}
100 kD
100 kD
LDHB
37 kD
37 kD
LDHA
50 kD
MV3
M233
HEMs
WM852
ATCC72
Lu1205
WM1158
TPF10-741
WM983-A
WM983-B
C32
Vemurafenib resistant
B-Raf WT
B-RafV600E
α-tubulin
