# Supplementary material for: Importance of glycolysis and oxidative phosphorylation in advanced melanoma
Source: Mol Cancer. 2012 Oct 9;11:76. doi: 10.1186/1476-4598-11-76 (PMC3537610; doi:10.1186/1476-4598-11-76)
Supplement: Additional file 3 — Figure S3. LDHA and HIF-1α expression in nevi and melanomas. (A-B, panels a) TMA cores comprised of nevi, and primary and metastatic melanoma tissue core, probed with antibody to LDHA or HIF-1α, and counterstained with hematoxylin. (A-B, panels b) 10X magnification of select TMA cores. [file 1476-4598-11-76-S3.pptx]

## Slide 1
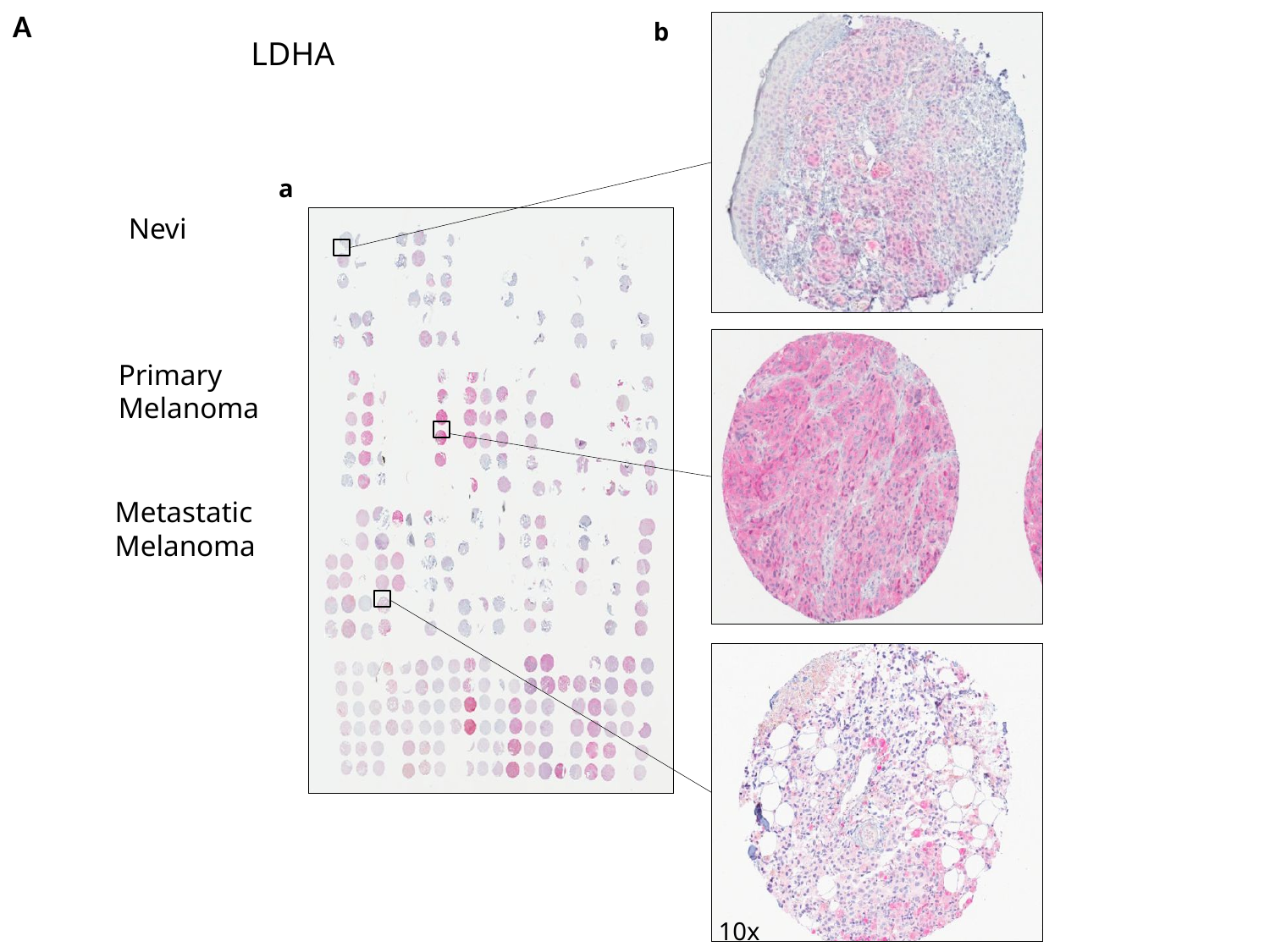

A
b
LDHA
a
Nevi
Primary
Melanoma
Metastatic
Melanoma
10x

## Slide 2
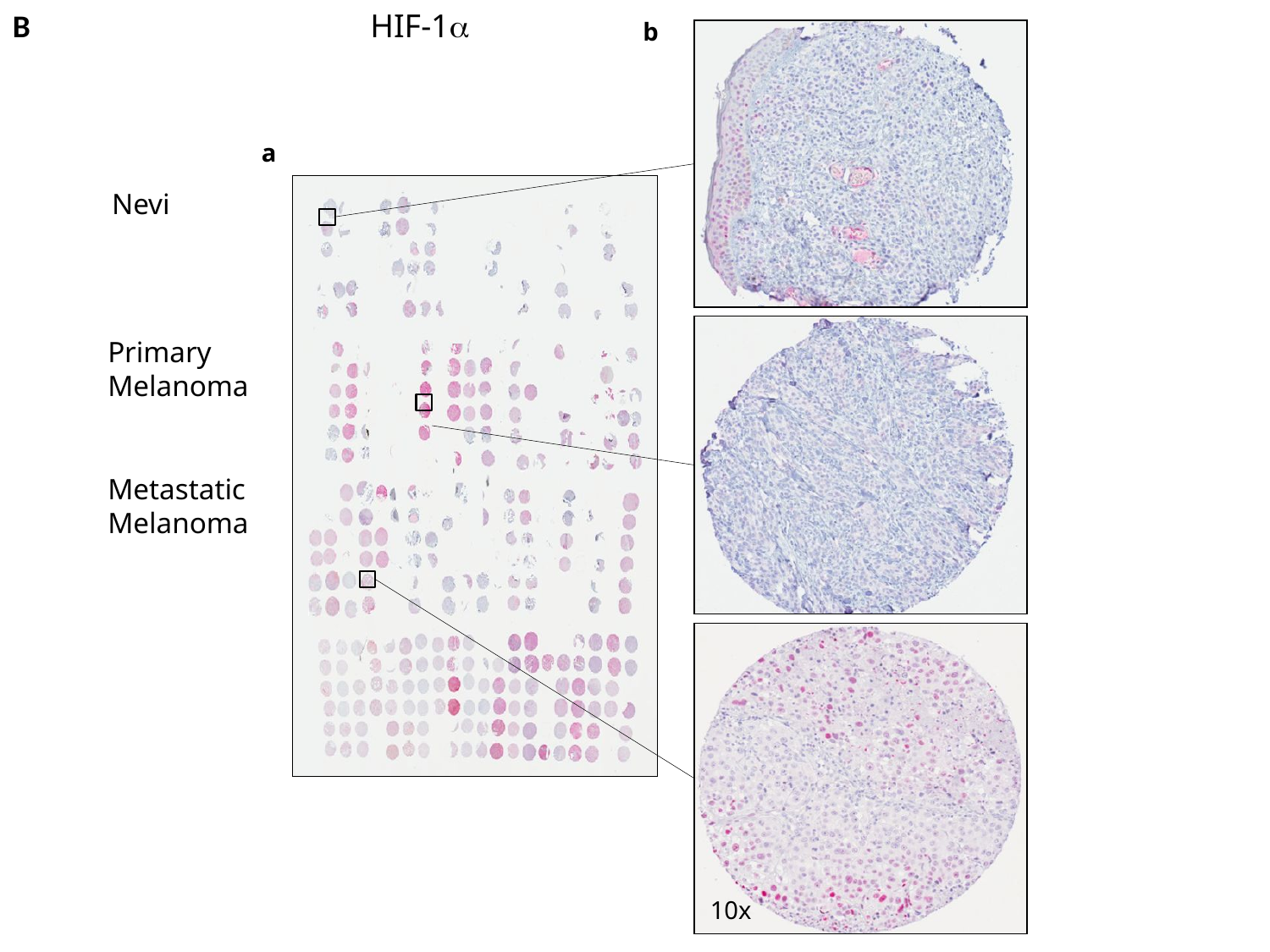

B
HIF-1α
b
a
Nevi
Primary
Melanoma
Metastatic
Melanoma
10x
