# Supplementary material for: Importance of glycolysis and oxidative phosphorylation in advanced melanoma
Source: Mol Cancer. 2012 Oct 9;11:76. doi: 10.1186/1476-4598-11-76 (PMC3537610; doi:10.1186/1476-4598-11-76)
Supplement: Additional file 4 — Figure S4. ATP5A1 and LDHB expression in nevi and melanomas. (A-B, panels a) TMA cores comprised of nevi, primary melanoma, and metastatic melanoma, probed with antibody to ATP5A1 or LDHB, and counterstained with hematoxylin. (A-B, panels b) 10X magnification of select TMA cores. [file 1476-4598-11-76-S4.pptx]

## Slide 1
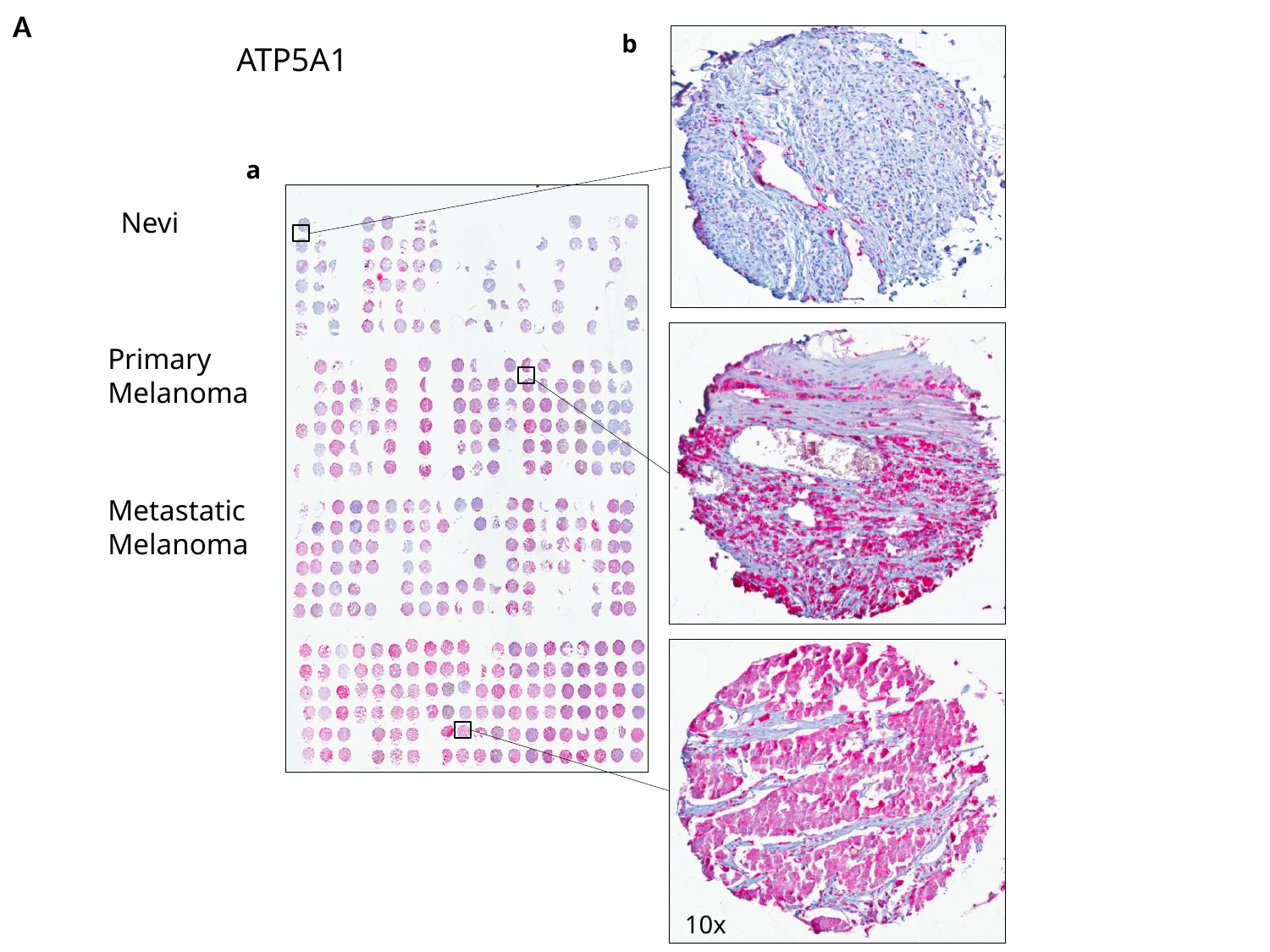

A
b
a
Nevi
Primary
Melanoma
Metastatic
Melanoma
10x
ATP5A1

## Slide 2
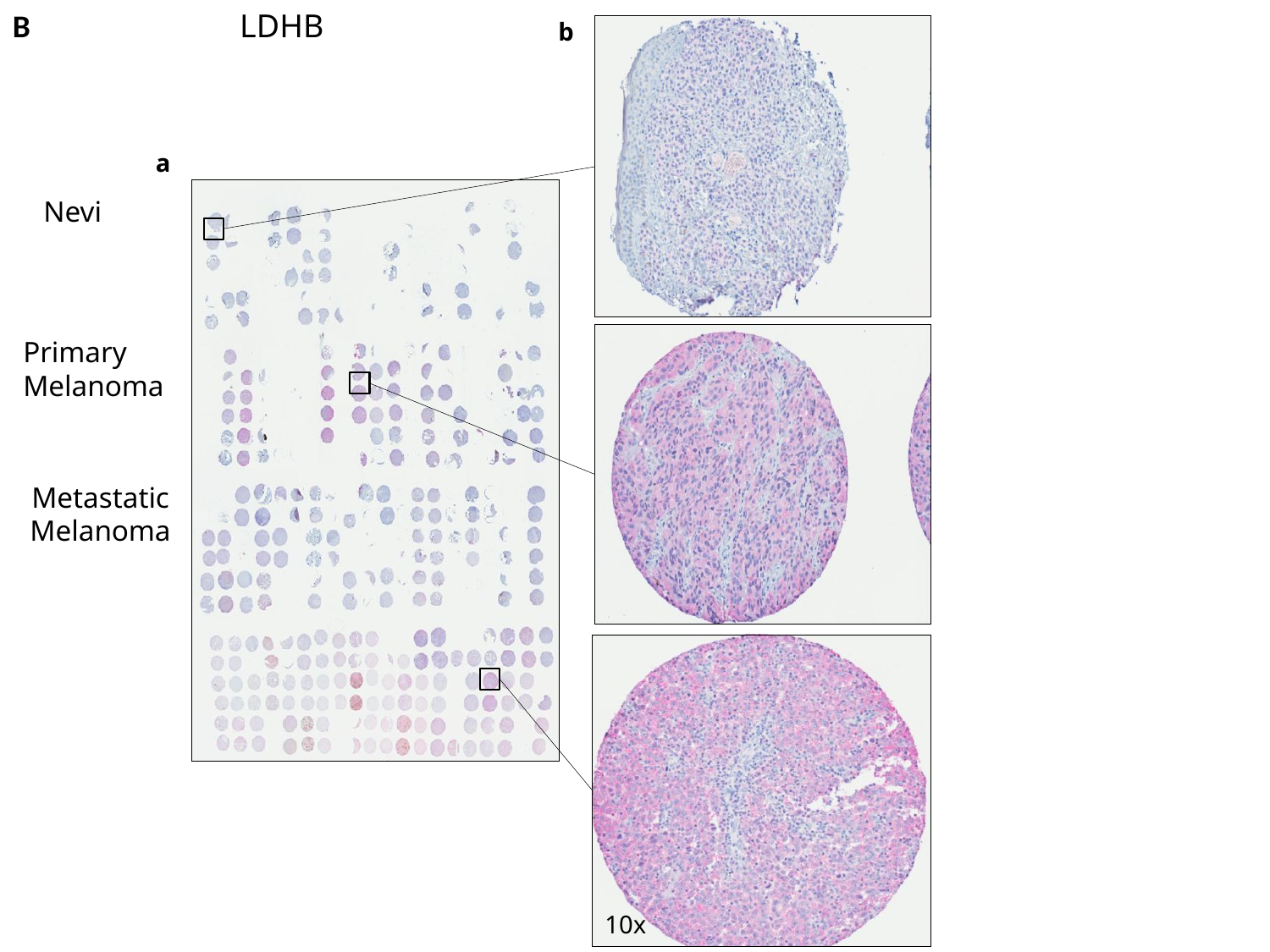

B
LDHB
b
a
Nevi
Primary
Melanoma
Metastatic
Melanoma
10x
