# Supplementary material for: Importance of glycolysis and oxidative phosphorylation in advanced melanoma
Source: Mol Cancer. 2012 Oct 9;11:76. doi: 10.1186/1476-4598-11-76 (PMC3537610; doi:10.1186/1476-4598-11-76)
Supplement: Additional file 5 — Figure S5. MCT1 and MCT4 expression in the nevus>melanoma TMA. The TMA study was performed as described in the legends to Additional files 3 and 4: Figures S3 and S4. [file 1476-4598-11-76-S5.pptx]

## Slide 1
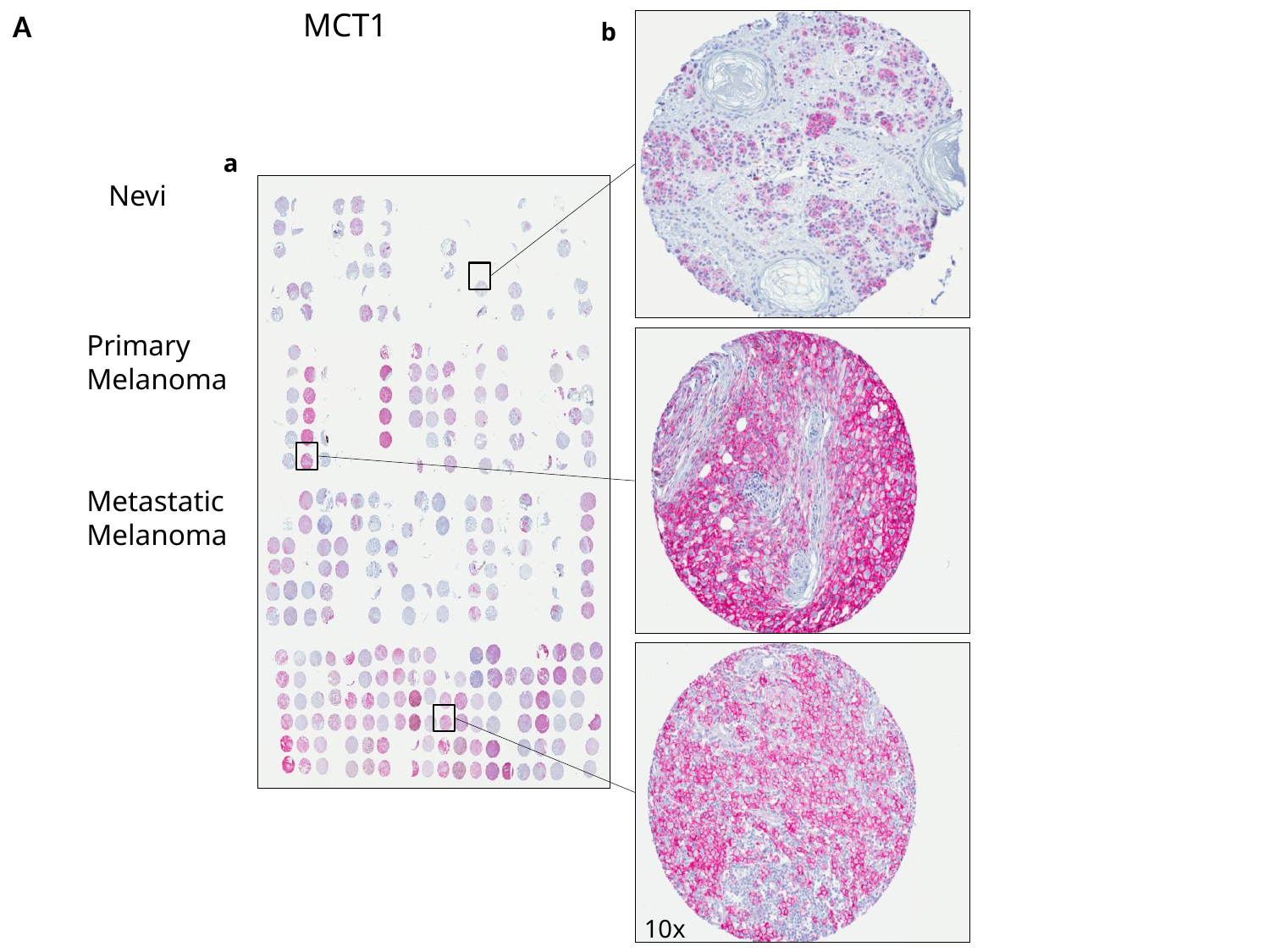

A
MCT1
b
a
Nevi
Primary
Melanoma
Metastatic
Melanoma
10x

## Slide 2
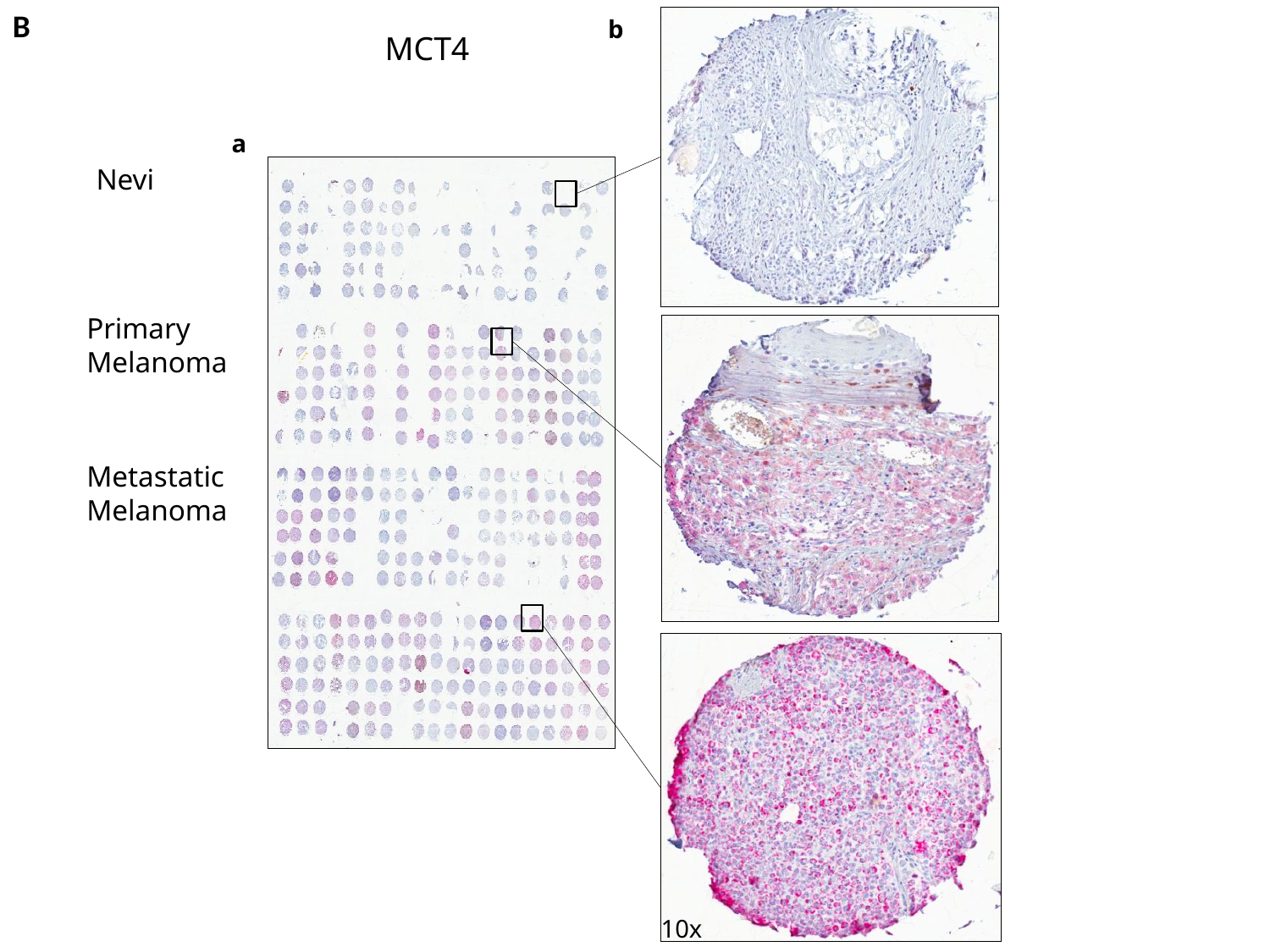

B
b
a
Nevi
Primary
Melanoma
Metastatic
Melanoma
10x
MCT4
